# Cross-sectional measures and modelled estimates of blood alcohol levels in UK nightlife and their relationships with drinking behaviours and observed signs of inebriation

**DOI:** 10.1186/1747-597X-5-5

**Published:** 2010-04-20

**Authors:** Mark A Bellis, Karen Hughes, Zara Quigg, Michela Morleo, Ian Jarman, Paulo Lisboa

**Affiliations:** 1 Centre for Public Health, Liverpool John Moores University, 5th Floor Kingsway House, Hatton Garden, Liverpool, L3 2AJ, UK; 2 Department of Mathematics and Statistics, Liverpool John Moores University, Byrom Street, Liverpool, L3 3AF, UK

## Abstract

**Background:**

Management of nightlife in UK cities focuses on creating safe places for individuals to drink. Little is known about intoxication levels as measuring total alcohol consumption on nights out is complicated by early evening interviews missing subsequent consumption and later interviews risking individuals being too drunk to recall consumption or participate at all. Here we assess mixed survey and modelling techniques as a methodological approach to examining these issues.

**Methods:**

Interviews with a cross sectional sample of nightlife patrons (n = 214) recruited at different locations in three cities established alcohol consumption patterns up to the point of interview, self-assessed drunkenness and intended drinking patterns throughout the remaining night out. Researchers observed individuals' behaviours to independently assess drunkenness. Breath alcohol tests and general linear modelling were used to model blood alcohol levels at participants' expected time of leaving nightlife settings.

**Results:**

At interview 49.53% of individuals regarded themselves as drunk and 79.43% intended to consume more alcohol before returning home, with around one in ten individuals (15.38% males; 4.35% females) intending to consume >40 units (equal to 400 mls of pure alcohol). Self-assessed drunkenness, researcher observed measures of sobriety and blood alcohol levels all correlated well. Modelled estimates for blood alcohol at time of going home suggested that 71.68% of males would be over 0.15%BAC (gms alcohol/100 mls blood). Higher blood alcohol levels were related to drinking later into the night.

**Conclusions:**

UK nightlife has used substantive health and judicial resources with the aim of creating safer and later drinking environments. Survey and modelling techniques together can help characterise the condition of drinkers when using and leaving these settings. Here such methods identified patrons as routinely getting drunk, with risks of drunkenness increasing over later nights. Without preventing drunkenness and sales to intoxicated individuals, extended drinking hours can simply act as havens for drunks. A public health approach to nightlife is needed to better understand and take into account the chronic effects of drunkenness, the damages arising after drunk individuals leave city centres and the costs of people avoiding drunken city centres at night.

## Background

In many countries, developing a safer nightlife has become synonymous with reducing violence, accidents and other immediate threats to health and well-being in town and city centres [1,2]. Safety initiatives often include elements such as high visibility policing, security staff located at late night transport points, improved street lighting, closed circuit television cameras and strict enforcement activity targeted at bars associated with alcohol-related crime [3,4]. Such measures aim to discourage the illegal and anti-social behaviours frequently associated with heavy drinking, but can allow such drinking to continue unabated. Thus, individuals who have drank heavily or who consider themselves drunk can often continue to participate in nightlife so long as they do not create a major disturbance, or until their increasing intoxication puts their own safety in obvious and immediate danger (e.g. they cannot walk) [5,6]. In contrast, less investment has focused on initiatives to prevent the underlying cause of many nightlife problems, i.e. drinking to intoxication. Sales regulations can prevent individuals buying alcohol when they are overtly intoxicated but these can be ignored by vendors due to factors including commercial pressure to sell alcohol, low awareness or personal responsibility by bar staff, and difficulties identifying and refusing service to drunk customers [7-9]. Campaigns targeting heavy drinkers can warn of the acute harms of drunkenness (e.g. sexual assault, injury) and provide harm reduction advice (e.g. encouraging consumption of non-alcoholic drinks alongside alcoholic ones), but typically such advice does not set or suggest any upper alcohol limits for a drinking session [10].

Managing rather than preventing drunkenness in nightlife places considerable pressures on public services through high cost policing [11,12] and treatment for those intoxicated or injured [13]. Moreover, the success of such management strategies is often measured by levels of violence and injury recorded in public nightlife settings [3,14,15]. Frequently, it ignores harms that may occur when people return to residential areas (e.g. subsequent public disturbances) or within individuals' homes (e.g. alcohol-related domestic violence, child abuse and fires [16-18]). Furthermore, dangers to drinkers' proximal health (e.g. alcohol-related asphyxia at home), employment (e.g. next day absenteeism and workplace injuries) and longer term well-being (e.g. alcohol-related liver disease) are also excluded from assessments of nightlife safety.

The UK has a well established culture of heavy drinking in nightlife settings [19]. Despite this, there is relatively little information available on either self-assessed, independently observed (i.e. observed sobriety) or biologically measured (i.e. blood alcohol level) drunkenness in nightlife environments [20,21]. The paucity of such information leads to no clear understanding of what constitutes drunkenness, the health dangers that getting routinely drunk represent, or how regulations to reduce drunkenness in night time environments (e.g. no sales to intoxicated individuals) might be implemented. While the UK has laws restricting alcohol sales to drunk individuals, these are rarely enforced [22]. Further, despite extending licensing hours to avoid binge drinking sessions just prior to bars closing, there is a lack of work examining changes in drunkenness (rather than crime or injury) in nightlife settings resulting from later opening hours [23]. Here, we have undertaken a study across three UK cities to explore self-assessed, independently observed and biologically measured drunkenness in nightlife patrons during their nights out. While other studies have used cross-sectional surveys combined with breath alcohol tests to explore relationships between blood alcohol levels, sobriety and alcohol consumption [20,21,24,25], interpreting such methods is complicated by early evening interviews missing subsequent consumption and later interviews risking individuals being too drunk to recall consumption or participate at all. Here we use direct empirical measures combined with modelling techniques to calculate the state of inebriation in which individuals are likely to return home and how this relates to drinking behaviours, demographics and the time at which people leave city centres.

## Methods

Three major city centres in the North West of England, each with a well developed nightlife, were utilised as study sites (Liverpool, Manchester and Chester). Teams of two researchers accompanied by a supervisor worked on Friday and Saturday nights in March and April 2009 between 8pm and 2am. Recruitment of participants used a structured approach with two teams working in parallel and using a series of different locations within each city for periods of one to two hours at a time (target sample n = 200). However despite sampling occurring across each city's nightlife areas, participants were not expected to be a representative sample but rather a prospective sample indicative of the range of individuals engaged in recreational drinking in nightlife settings. Participants completed a short anonymous questionnaire and undertook a breath alcohol test (BrAT). The questionnaire examined: quantities of alcohol consumed to the point of survey (by type of beverage); whether individuals had preloaded before going out that night (e.g. drank alcohol at their own or a friend's home); age; height; and whether respondents felt drunk or believed they were above the legal UK limit for drink-driving (80 milligrams of alcohol per 100 millilitres of blood; 0.08%BAC; blood alcohol concentration). The survey also explored how many hours had passed since the beginning of their drinking session, the time since they last ate a meal, the time at which they would typically expect to leave the nightlife setting and how much more alcohol they intended to consume before leaving. All questionnaires were completed by researchers on behalf of participants through an interview process.

### Participant recruitment

All individuals out for recreational purposes and drinking alcohol were eligible for inclusion in the survey. Of those individuals approached by researchers, all identified that they fulfilled these criteria and therefore none were rejected at that stage. However for the purposes of safety, and to accommodate ethics relating to informed consent, individuals showing severe signs of inebriation were excluded. To assess levels of drunkenness, all potential participants were visually assessed by researchers through a tool incorporating measures used by police and in previous studies [20]. Prior to being approached, individuals were monitored for steadiness on their feet (staggering, swaying) and loud or aggressive talking (Likert scale; 1 = none to 5 = strong signs). Individuals scoring four or more in any category were not approached. For those approached and agreeing to participate, further measures (difficulty focusing, slurring words, incoherent speech, glazed eyes and close talking distance) were assessed on the same scale throughout the interview process. Calculating the total number of individuals excluded due to severe inebriation was not possible as no record was kept of those individuals who were so inebriated (e.g. could not walk) that they would clearly fail on any sobriety assessment. Researchers also made visual assessments of participants' build on a scale of 1 to 5 (1 = very slight, 5 = heavy build). Researchers were trained in the application of these assessments in order to improve grading consistency. A total of 271 eligible individuals were approached for the study, of which 57 (21.03%) declined to take part before the purpose of the research was explained to them. None subsequently refused once the study had been explained. Overall, a final sample of 214 took part in the survey (n = 111, 65, 38 in Liverpool, Manchester and Chester respectively).

### Measuring blood alcohol concentrations

Participants were recruited and interviewed in streets outside nightlife areas and around transport points (e.g. bus and taxi ranks). A BrAT was conducted on all participants using the Lion Alcometer^® ^500 Breath Alcohol Kit, a variation of the model used by UK police [26] and other law enforcement agencies. To comply with BrAT requirements, the study process was designed to ensure sufficient time had passed (20 minutes) for any alcohol in the participants' mouths to have absorbed prior to breath testing, and participants were requested not to smoke during interview [27]. Each participant was provided with their own mouthpiece, which was discarded safely once used. The analytical response time of the test is typically within 30 seconds and BrAT test scores were immediately provided to participants, as well as being recorded by researchers. For the purposes of analysis BrAT results were converted into %BAC (according to established UK ratios) as this is a more commonly used and legally referenced measure [28]. Reported alcohol products consumed were converted into standard UK units (1 unit = 8 grams or 10 ml of pure alcohol) using published figures for alcohol contents (e.g. single shot of spirits = 1 unit; bottle of lager = 1.5 units; standard glass of wine = 2 units [29]).

### Modelling final blood alcohol

Most individuals surveyed (79.43%) had not completed their planned alcohol consumption for that night (and if they had may have been too intoxicated to fulfil inclusion criteria). Significant relationships between %BAC and all variables measured at the time of interview, e.g. demographics, body size, units consumed and drinking rate, were estimated with General Linear Models (GLM) [30]. All continuous variables were log transformed and in the final model no demographic or body size variables were significant (Table 1). Individuals had already provided details of how much longer they would expect to remain out drinking and their estimated additional alcohol consumption over the remainder of their night out. By adding these to measures of hours drinking, units consumed and hours since having eaten a meal at time of interview, new estimated final total units consumed, total hours drinking, average drinking rate and hours since having eaten a meal for their entire session (i.e. up to point of expected home time) were calculated. For each individual these data were then used within the model to estimate final %BAC at their expected time of departing the nightlife setting.

**Table 1:** General linear model for prediction of blood alcohol levels

Model items	Estimate	SE	F	P
Intercept	-1.8844	0.07	631.7	<0.001
Log units per hour	0.6369	0.09	53.47	<0.001
Log hours drinking	0.7333	0.07	112.8	<0.001
Log hours since ate	0.1595	0.05	9.087	<0.005

Age, sex, height, build, city and preloading were also included in the stepwise model but only hours drinking, drinking rate and hours since eating a meal were significantly related to %BAC at interview. The final model accounted for 40.02% of the variance. Degrees of freedom: Model = 3; Intercept = 1; Log units per hour = 1, Log hours drinking = 1, Log hours since ate = 1; Error = 207. SE = standard error

### Statistical analyses

All data were entered into, and analysed using, SPSS (V15). Statistical analyses utilised chi-square, ANOVA and GLM. Research was reviewed and passed by Liverpool John Moores University Research Ethics Committee.

## Results

Samples did not differ between study sites in mean number of hours drinking, total units consumed, units consumed per hour (units/hr) or mean %BAC at time of interview (Additional file 1). Mean values for males were higher than females for each measure (Additional file 1). Those who reported preloading before arriving in city centres had consumed more units of alcohol although differences in %BAC (between those who did and did not preload) just failed to reach significance (Additional file 1). However, %BAC, hours drinking and total units consumed were positively associated with self-reported feeling drunk, while %BAC and units consumed were also associated with having recently eaten a meal (specifically in the last four hours; Additional file 1). Build and age were not related to any measures of drinking behaviour or %BAC. While height was related to number of units consumed, units/hr and %BAC, this was primarily due to its relationship with sex. Thus, when analysed separately by gender, no measures of drinking behaviours were significantly related to height, and %BAC only differed by height category in females (ANOVA, F_3,90 _= 2.857, P < 0.05). All individuals, regardless of %BAC, were informed about their BrAT results and whether the reading was above or below UK legal driving limits. Only 3.55% stated that they would drink less as a result of knowing their %BAC (mean %BAC = 0.15) with 24.87% saying they would drink more (mean %BAC = 0.12) and 71.57% saying it would have no effect (mean %BAC = 0.11; %BACs were not significantly different between groups, ANOVA, F_2,194 _= 0.922, P = 0.399).

Additional file 2(a-d) compares the distribution of units consumed, hours drinking and units/hr as well as %BAC at time of interview (actual) and at planned time of leaving city centres (home time) for males and females separately. For both sexes there was no significant difference between drinking rate during the period up to being interviewed and the rate estimated for the whole evening up until home time (Additional file 2c). However, in both males and females estimated %BAC was higher at home time compared with measured %BAC at time of interview (Additional file 2d). Across all measures of drinking behaviour and %BAC, males' home time estimates were significantly different to females', with higher %BACs, units drank, hours drinking and units/hr in males (Additional file 2a-d). Mean predicted %BAC for males at home time was 0.19 (95%CIs, 0.17-0.20) and for females 0.13 (95%CIs, 0.11-0.14) and total units consumed were 27.43 (95%CIs, 24.68-30.18) and 16.17 (95%CIs, 13.84-18.49) respectively. For those who preloaded (vs those who did not preload) the estimated total time drinking (mean hours, 9.40 vs 7.77, ANOVA, F_1,208 _= 8.47, P < 0.005), total units (mean units, 25.97 vs 18.63, ANOVA, F_1,207 _= 14.77, P < 0.001), units/hr over the whole drinking session (mean units/hr, 2.95 vs 2.38, ANOVA, F_1,203 _= 6.91, P < 0.01) and expected %BAC at home time (mean %BAC, 0.18 vs 0.14, ANOVA, F_1,201 _= 16.32, P < 0.001) were all significantly higher.

Levels of %BAC at interview were strongly related to observational sobriety measures. Proportions of individuals showing signs of each drunkenness measure (score ≥ 2) increased significantly with measured %BAC (Additional file 3). Thus, only 15% of those with a %BAC of ≤ 0.05 were showing signs of unsteadiness on their feet compared with all of those with a %BAC >0.25. Similarly figures for slurring speech during interviews rose from 22.50% to 100% respectively. Furthermore, self-assessed drunkenness was strongly related to %BAC. Half (49.53%) of participants reported feeling drunk at interview, increasing from 10.00% of those with %BAC ≤ 0.05 to 83.33% of those with %BAC >0.25 (Additional file 3). Self-assessed drunkenness also correlated well with researcher-observed measures with, for instance, 71.70% of those assessed as unsteady on their feet self-reported as drunk (Additional file 3).

**Figure 1:**
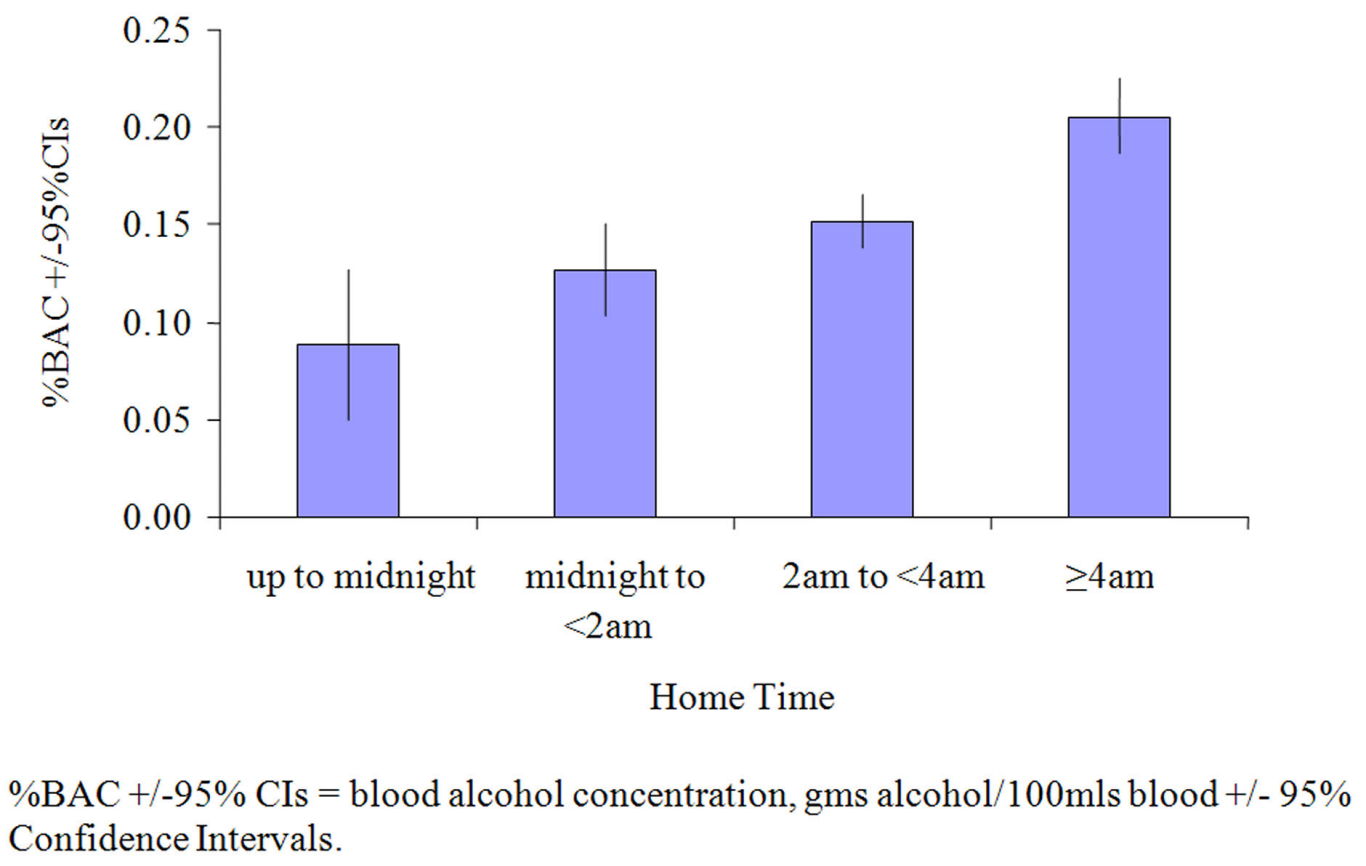
**Relationship between expected home time and modelled blood alcohol concentration at home time**. %BAC = blood alcohol concentration, gms alcohol/100 mls blood.

Finally, the relationship between participants' expected home time and predicted %BAC at that time was examined. There was a strong positive correlation between %BAC and time leaving nightlife, increasing from a mean of 0.09%BAC in those intending to leave before midnight to 0.21%BAC in those intending to leave at or after 4am (Figure 1).

## Discussion

In order to examine relationships between alcohol consumption and levels of inebriation in patrons of UK nightlife, a cross-sectional survey of drinkers' %BAC and drinking behaviours was undertaken in three UK cities. Individuals who were already severely inebriated [21,31] were excluded. This may have resulted in conservative estimates of drunkenness and alcohol consumption. However, our methods included many individuals who assessed themselves as being drunk and who would become drunk later in the night. Moreover, this did not affect within-individual comparisons of drinking behaviours with observed, self-assessed and biologically measured (BrAT) drunkenness. Consequently, GLMs were employed to calculate the %BAC of participants at their point of departure from nightlife centres, frequently after they would have drunk substantially more alcohol (Additional file 2). The explanatory variables in this observational study were restricted to demographic and self-reported behavioural information, from which GLMs explained 40% of the variation in %BAC. However, the impact of the unexplained variance is likely to be mitigated by comparisons and conclusions being based on mean %BACs rather than calculations for any individual. Further, this methodology: achieved good compliance; is likely to have allowed better recall than interviewing more inebriated people at the end of the night [31]; and resulted in reported consumption consistent with other studies of similar populations [5]. Unlike in other studies, our final model did not improve from the inclusion of sex or body size and even forced inclusion of these measures did not improve the variance explained [32-34]. However, the model was developed using data from the same individuals to which it was subsequently applied and was bespoke to real drinking environments rather than based on laboratory like conditions. Thus, while other generic models for calculating %BAC from consumption are available [33], when used in comparable drinking environments they have resulted in poorer predictions of %BAC (e.g. only around 20% of variance explained [35]). Importantly, when using the model predictions of %BACs at home time, the vast majority of home time (estimated) values for hours drinking (97.1%), drinking rates (100%), hours since at a meal (97.1%) and %BAC (99.5%) were within the observed range at interview. Therefore the model was not extrapolating substantially beyond its training values. Our %BAC measurements also relied on the appropriate use of the BrATs. In particular, a period of 20 minutes is recommended between last drink and breath analysis [27]. Consequently, our study was designed to maximise time between drinking and BrAT, with participants approached outside of drinking establishments, and then introduced to the study and interviewed before %BAC was measured. Finally, there was no control of whether, post-interview, individuals would consume another meal before home time. However, individuals typically seek takeaway meals or other food at the end of the evening and food consumption appears to have made only a relatively small difference to %BAC (table 1) compared with other factors in our model.

Even at interview and with study criteria excluding those showing strong signs of drunkenness, 49.53% of respondents assessed themselves as being drunk. At least in our sample, drunkenness was a typical part of nights out rather than, as sometimes suggested, limited to a few individuals [36]. At home time, modelling suggests %BACs will be considerably higher (Additional file 2d) and drunkenness will be the rule rather than the exception. Given the patterns of alcohol consumption identified this is not surprising. By home time, 10.53% of individuals (15.38% males; 4.35% females; Additional file 2a) intended to have drunk more than 40 units. In fact, even at interview 20.00% of males and 21.28% of females had drank more than the weekly alcohol limits (21 units, males; 14 units, female [37]) recommended by the UK government prior to the introduction of daily recommended limits, and at home time these figures were estimated to rise to 60.68% and 44.57% respectively. Excess alcohol was often consumed over long drinking periods. For males 21.18% of individuals were expecting to have been drinking for more than 12 hours before returning home. Time drinking, total units consumed and, to a lesser extent, units consumed per hour were positively related to preloading and resulted in preloaders having a significantly higher predicted %BAC at home time.

As well as effects relating to preloading, our results are consistent with extensions to licensing hours contributing to a higher prevalence of drunkenness (Figure 1). In 2005, licensing regulations in England and Wales changed to allow alcohol to be sold 24 hours a day [38]. Although most on- and off-licence establishments have not adopted 24 hour opening, many have extended their opening hours [39]. Here, those individuals intending to utilise the later hours were also most likely to have the highest %BACs (Figure 1). While our sample could not be considered representative of all UK nightlife users, results would at least support the hypothesis that later opening hours can increase inebriation and our methodology provides a mechanism for subsequent tests of this relationship. Already, police and health resources are stretched into early morning hours to allow drunkenness to progress in relative safety and to respond when incidents occur [12,40]. Importantly, high visibility policing and easily accessible emergency health care may actually encourage individuals to get drunk in the knowledge that the immediate risks associated with drunkenness are substantially reduced [41,42].

As technologies such as those employed in this study to measure %BAC (BrAT) become more accessible and affordable they may create an additional pressure to consume more alcohol. Only 3.55% of individuals said they would reduce their drinking once informed of their %BAC while nearly one in four individuals thought they would drink more. While this phenomenon needs more study, individuals in the UK can feel that an important feature of a night out drinking is to become drunk. A measured %BAC close to, or even under, the legal driving limit may appear to some drinkers as inconsistent with such an objective and consequently provide an incentive to drink more. Commercial use of BrATs to encourage individuals to drink more has already been attempted in some bars in the UK [43] and similar problems have been seen elsewhere [3]. Such tests also pose a danger for drink-driving. In our sample, of those below the legal maximum %BAC for driving in the UK (0.08%BAC), 18.31% considered themselves drunk (Additional file 3) but on BrAT realised that they could still legally drive a car. Consequently, easy access to BrAT in night time environments may increase the risk of those who feel drunk at lower blood alcohol levels attempting to drive home in the knowledge they are under the legal blood alcohol limit. Self-reported drunkenness was less common (10%, Additional file 3) in those within the typical European driving limit (up to 0.05%BAC) than in those between 0.05%BAC and the UK limit, where 29.03% considered themselves drunk (Additional file 3). Thus, moves to a lower legal %BAC limit for driving in the UK [44,45] could help prevent drunks driving, with less people who feel drunk identifying (e.g. through BrAT) that they are legally allowed to drive.

In our sample, half (51.16%) of those who considered themselves drunk at interview intended to consume more alcohol that night. In the UK and elsewhere it is illegal to sell alcohol to those who are drunk. However, research suggests that such laws are often ignored through, for example, commercial pressures to sell alcohol, low awareness and responsibility among bar servers, and difficulties recognising and refusing service to drunks [7-9]. Importantly, those breaking the law are rarely identified and penalised; in 2007 available data show just one individual (out of just seven proceeded against) was found guilty of selling alcohol to a drunk person in England and Wales, with 81 penalty notices for disorder (PNDs) issued for the same offence [22] (PNDs can be issued by police for certain alcohol-related offences, carrying a fine of £50-£80 to the offender). Findings here identify that a series of relatively simple behavioural observations are strongly correlated with drunkenness and %BAC (Additional file 3). Of those both unsteady on their feet and with a %BAC over 0.20, 83.33% self-assessed as drunk. Our results, and those of others, suggest that simple diagnostic observations of drunkenness (with or without BrAT measures) could be developed for nightlife settings and implemented by trained door and bar staff [20]. Along with such training, measures would have to be implemented to ensure staff feel confident about their own safety when refusing entry to a premises or service to a drunk and potentially aggressive individual. Critically however, such measures run counter to commercial interests. Thus, much of the alcohol sold in night time environments is consumed by those who are already drunk and, to a large extent, the economic viability of many late night businesses in the UK relies on patronage by drunks. Consequently, measures to reduce alcohol sales to drunk individuals are unlikely to be adopted unless made mandatory. However, properly implemented such measures could substantially reduce the number of drunk individuals who continue to access alcohol in nightlife environments and result in fewer highly inebriated individuals leaving nightlife environments early in the morning or requiring health and judicial attention.

## Conclusions

Cities in the UK have adopted costly nightlife strategies aimed at protecting patrons from immediate alcohol-related harms, and controlling violence and other anti-social behaviour. Implementing safety measures in nightlife environments is crucial to protecting public health, yet without reasonable efforts to reduce nightlife alcohol consumption such measures may simply result in safer environments for drunks. Typically, assessment of nightlife uses police and emergency department data but ignores the underlying trends in excessive alcohol consumption. Here we have explored a novel method to expose underlying drinking behaviours and their progressive relationship with drunkenness during nights out. In particular, this approach provides a method for examining the more extreme levels of alcohol consumption associated with drinking into the early hours of the morning without either exposing researchers to highly inebriated consumers with poor recall or relying on estimations based on data from largely artificial, controlled and unrelated environments [46]. Although only preliminary, results using this methodology suggest, for instance, that preloading (typically with cheaper alcohol [5,47]) and drinking later into the night may be associated with higher levels of drunkenness in city centres. Initiatives informed by such intelligence may not only reduce acute harms and anti-social behaviours but also allow many adults who deliberately avoid heavy drinking cultures to re-engage with their city centres at night [48].

## Competing interests

The authors declare that they have no competing interests.

## Authors' contributions

MAB designed the study, analysed the data and wrote the manuscript. KH and ZQ contributed to the design of the study, collected the data, assisted with data analyses and helped to draft and edit the manuscript. MM contributed to the design of the study, data collection and drafting the manuscript. IJ and PL assisted with data analyses and edited the manuscript. All authors read and approved the final manuscript.

## Supplementary Material

Additional file 1 Sample characteristics, drinking behaviours and blood alcohol levels at interview. Click here for file

Additional file 2 Comparisons of drinking behaviour and blood alcohol concentrations at point of interview with estimated drinking behaviour over entire evening (up to estimated home time) and modelled blood alcohol concentration at intended home time. Click here for file

Additional file 3 Relationship between individuals' blood alcohol concentration, self-assessment as drunk and researcher-assessed signs of drunkenness. Click here for file
