# Supplementary material for: Cross-sectional measures and modelled estimates of blood alcohol levels in UK nightlife and their relationships with drinking behaviours and observed signs of inebriation
Source: Subst Abuse Treat Prev Policy. 2010 Apr 20;5:5. doi: 10.1186/1747-597X-5-5 (PMC2873259; doi:10.1186/1747-597X-5-5)
Supplement: Additional file 1 — Sample characteristics, drinking behaviours and blood alcohol levels at interview. [file 1747-597X-5-5-S1.DOC]

**Table 2. Sample characteristics, drinking behaviours and blood alcohol levels at interview**

|  |  |  | **Hours drinking** | | | **Units consumed** | | | **Units per hour** | | | **%BAC** | | |
| --- | --- | --- | --- | --- | --- | --- | --- | --- | --- | --- | --- | --- | --- | --- |
|  | | n | Mean | SD | P | Mean | SD | P | Mean | SD | P | Mean | SD | P |
| Age | *16-20* | 78 | 4.90 | 3.67 | 0.338 | 13.63 | 9.58 | 0.489 | 3.45 | 2.65 | 0.083 | 0.11 | 0.07 | 0.246 |
|  | *21-25* | 65 | 5.66 | 3.83 |  | 14.82 | 11.59 |  | 2.98 | 2.06 |  | 0.13 | 0.07 |  |
|  | *26+* | 71 | 5.66 | 3.42 |  | 12.82 | 8.06 |  | 2.64 | 1.71 |  | 0.12 | 0.07 |  |
| Sex | *male* | 120 | 5.89 | 3.90 | <0.05 | 16.21 | 10.72 | <0.001 | 3.44 | 2.62 | <0.005 | 0.13 | 0.08 | <0.005 |
|  | *female* | 94 | 4.73 | 3.19 |  | 10.55 | 7.34 |  | 2.52 | 1.40 |  | 0.10 | 0.06 |  |
| Location | *Liverpool* | 111 | 5.77 | 4.13 | 0.251 | 14.76 | 10.98 | 0.163 | 3.10 | 2.00 | 0.32 | 0.12 | 0.07 | 0.854 |
|  | *Manchester* | 65 | 5.04 | 2.89 |  | 11.85 | 8.14 |  | 2.73 | 2.59 |  | 0.11 | 0.08 |  |
|  | *Chester* | 38 | 4.82 | 3.20 |  | 13.91 | 8.28 |  | 3.38 | 2.08 |  | 0.12 | 0.06 |  |
| Build | *slight* | 98 | 5.22 | 3.05 | 0.799 | 13.86 | 10.18 | 0.948 | 2.86 | 1.76 | 0.544 | 0.12 | 0.07 | 0.269 |
|  | *average* | 77 | 5.57 | 3.94 |  | 13.40 | 8.62 |  | 3.21 | 2.84 |  | 0.12 | 0.08 |  |
|  | *heavy* | 38 | 5.24 | 4.34 |  | 13.86 | 11.22 |  | 3.17 | 1.84 |  | 0.10 | 0.06 |  |
| Height | *<=160* | 22 | 4.49 | 2.82 | 0.183 | 10.45 | 7.30 | <0.01 | 2.39 | 1.01 | <0.05 | 0.07 | 0.04 | <0.005 |
| (centimetres) | *>160-170* | 53 | 5.16 | 3.19 |  | 11.33 | 8.44 |  | 2.50 | 1.50 |  | 0.12 | 0.06 |  |
|  | *>170-180* | 71 | 5.16 | 3.78 |  | 14.10 | 10.72 |  | 3.12 | 1.89 |  | 0.11 | 0.07 |  |
|  | *>180* | 66 | 6.17 | 3.99 |  | 16.61 | 9.82 |  | 3.37 | 2.26 |  | 0.14 | 0.08 |  |
| Preloaded | *no* | 100 | 4.99 | 3.41 | 0.141 | 11.59 | 8.82 | <0.005 | 2.81 | 2.35 | 0.151 | 0.11 | 0.07 | 0.071 |
|  | *yes* | 114 | 5.72 | 3.81 |  | 15.60 | 10.24 |  | 3.24 | 2.08 |  | 0.12 | 0.07 |  |
| Feel drunk | *no* | 108 | 4.50 | 3.60 | <0.001 | 11.06 | 9.13 | <0.001 | 2.98 | 2.47 | 0.714 | 0.09 | 0.06 | <0.001 |
|  | *yes* | 106 | 6.28 | 3.47 |  | 16.44 | 9.73 |  | 3.09 | 1.93 |  | 0.15 | 0.07 |  |
| Eaten meal in | *no* | 168 | 5.58 | 3.52 | 0.130 | 14.53 | 9.84 | <0.05 | 3.01 | 1.88 | 0.738 | 0.13 | 0.07 | <0.001 |
| last 4 hours | *yes* | 46 | 4.66 | 4.04 |  | 10.79 | 9.11 |  | 3.14 | 3.19 |  | 0.08 | 0.07 |  |

Analyses used ANOVA. %BAC = blood alcohol concentration, gms alcohol/100mls blood. SD = Standard deviation.
