# Supplementary material for: Cross-sectional measures and modelled estimates of blood alcohol levels in UK nightlife and their relationships with drinking behaviours and observed signs of inebriation
Source: Subst Abuse Treat Prev Policy. 2010 Apr 20;5:5. doi: 10.1186/1747-597X-5-5 (PMC2873259; doi:10.1186/1747-597X-5-5)
Supplement: Additional file 2 — Comparisons of drinking behaviour and blood alcohol concentrations at point of interview with estimated drinking behaviour over entire evening (up to estimated home time) and modelled blood alcohol concentration at intended home time. [file 1747-597X-5-5-S2.DOC]

**Table 3 – Comparisons of drinking behaviour and blood alcohol concentrations at point of interview with estimated drinking behaviour over entire evening (up to estimated** home time) and modelled blood alcohol concentration at intended home time

| **a** | **Units drank** |  | **n** | **1-5** | **>5-10** | **>10-20** | **>20-30** | **>30-40** | **>40** | **P^$^** | **P^** |
| --- | --- | --- | --- | --- | --- | --- | --- | --- | --- | --- | --- |
|  | Male | *Interview (%)* | 117 | 9.40 | 23.93 | 42.74 | 13.68 | 5.13 | 5.13 |  |  |
|  | *Estimated home time (%)* | | 117 | 1.71 | 6.84 | 27.35 | 29.06 | 19.66 | 15.38 | <0.001 |  |
|  | Female | *Interview (%)* | 92 | 22.83 | 41.30 | 28.26 | 4.35 | 2.17 | 1.09 |  |  |
|  | *Estimated home time (%)* | | 92 | 10.87 | 26.09 | 32.61 | 21.74 | 4.35 | 4.35 | <0.001 | <0.001 |
| **b** | **Hours drinking** | |  | **>0-2** | **>2-4** | **>4-8** | **>8-12** | **>12-18** | **>18** |  |  |
|  | Male | *Interview (%)* | 118 | 14.41 | 27.97 | 32.20 | 16.10 | 9.32 | 0.00 |  |  |
|  | *Estimated home time (%)* | | 118 | 0.85 | 6.78 | 37.29 | 33.90 | 16.10 | 5.08 | <0.001 |  |
|  | Female | *Interview (%)* | 92 | 16.30 | 34.78 | 41.30 | 3.26 | 3.26 | 1.09 |  |  |
|  | *Estimated home time (%)* | | 92 | 0.00 | 10.87 | 64.13 | 19.57 | 3.26 | 2.17 | <0.001 | <0.001 |
| **c** | **Units per hour** | |  | **<=1** | **>1-2** | **>2-3** | **>3-4** | **>4-6** | **>6** |  |  |
|  | Male | *Interview (%)* | 115 | 1.74 | 26.96 | 32.17 | 16.52 | 13.04 | 9.57 |  |  |
|  | *Estimated home time (%)* | | 115 | 1.74 | 26.09 | 35.65 | 19.13 | 13.91 | 3.48 | 0.587 |  |
|  | Female | *Interview (%)* | 90 | 8.89 | 37.78 | 27.78 | 15.56 | 6.67 | 3.33 |  |  |
|  | *Estimated home time (%)* | | 90 | 14.44 | 38.89 | 28.89 | 11.11 | 5.56 | 1.11 | 0.703 | <0.001 |
| **d** | **Blood alcohol concentration** | |  | **<=0.05** | **>0.05-0.08** | **>0.08-0.15** | **>0.15-0.20** | **>0.20-0.25** | **>0.25** |  |  |
|  | Male | *Interview (%)* | 113 | 16.81 | 13.27 | 30.97 | 18.58 | 15.04 | 5.31 |  |  |
|  | *Estimated home time (%)* | | 113 | 3.54 | 3.54 | 21.24 | 31.86 | 21.24 | 18.58 | <0.001 |  |
|  | Female | *Interview (%)* | 90 | 23.33 | 15.56 | 42.22 | 14.44 | 4.44 | 0.00 |  |  |
|  | *Estimated home time (%)* | | 90 | 7.78 | 10.00 | 53.33 | 22.22 | 2.22 | 4.44 | <0.01 | <0.001 |

For each gender, the table compares recorded drinking behaviour (a-c) for the evening up to the point of interview with estimates of the same behaviours for the whole evening (up to estimated home time). Individuals’ post-interview drinking behaviour is calculated from their questionnaire answers. Calculation of blood alcohol concentrations at home time (d) uses the same questionnaire information and the model parameters presented in Table 1. P^$^ compares values within sex between at interview and at end of evening. P^ compares values between sexes at expected home time from city centres. All statistics are based on chi-square tests. Sample is limited to individuals with values for both interview and home times. See methodology for calculations of drinking behaviours up to home times and blood alcohol at home time.
