# Supplementary material for: Cross-sectional measures and modelled estimates of blood alcohol levels in UK nightlife and their relationships with drinking behaviours and observed signs of inebriation
Source: Subst Abuse Treat Prev Policy. 2010 Apr 20;5:5. doi: 10.1186/1747-597X-5-5 (PMC2873259; doi:10.1186/1747-597X-5-5)
Supplement: Additional file 3 — Relationship between individuals' blood alcohol concentration, self-assessment as drunk and researcher-assessed signs of drunkenness. [file 1747-597X-5-5-S3.DOC]

**Table 4. Relationship between individuals’ blood alcohol concentration, self-assessment as drunk and researcher-assessed signs of drunkenness**

| **Signs of  drunkenness^$^** | **Blood Alcohol Concentration** | | | | | | | **Self-assessed as drunk** | | |
| --- | --- | --- | --- | --- | --- | --- | --- | --- | --- | --- |
|  | ***<=0.05*** | ***>0.05-0.08*** | ***>0.08-0.15*** | ***>0.15-0.20*** | ***>0.20-0.25*** | ***>0.25*** | **P^** | **No** | **Yes** | **P** |
| n | 40 | 31 | 77 | 36 | 23 | 6 |  | 108 | 106 |  |
| *Unsteady on feet (%)* | 15.00 | 45.16 | 59.74 | 75.00 | 78.26 | 100.00 | <0.001 | 38.89 | 71.70 | <0.001 |
| *Talking loud/aggressive (%)* | 15.00 | 38.71 | 55.84 | 75.00 | 73.91 | 83.33 | <0.001 | 39.81 | 63.21 | <0.001 |
| *Swaying (%)* | 20.00 | 54.84 | 57.14 | 72.22 | 82.61 | 100.00 | <0.001 | 43.52 | 69.81 | <0.001 |
| *Slurring words (%)* | 22.50 | 51.61 | 59.74 | 61.11 | 82.61 | 100.00 | <0.001 | 42.59 | 67.92 | <0.001 |
| *Incoherent speech (%)* | 7.50 | 35.48 | 48.05 | 63.89 | 78.26 | 83.33 | <0.001 | 32.41 | 59.43 | <0.001 |
| *Glazed eyes (%)* | 32.50 | 70.97 | 70.13 | 72.22 | 82.61 | 100.00 | <0.001 | 55.56 | 75.47 | <0.005 |
| *Close talking distance (%)* | 27.50 | 54.84 | 53.25 | 61.11 | 78.26 | 100.00 | <0.001 | 44.44 | 63.21 | <0.01 |
| *Difficult focusing (%)* | 15.00 | 41.94 | 45.45 | 66.67 | 78.26 | 83.33 | <0.001 | 33.33 | 61.32 | <0.001 |
| Self-assessed as drunk | 10.00 | 29.03 | 62.34 | 63.89 | 73.91 | 83.33 | <0.001 |  |  |  |

^$^>1 on the Likert scale = some evidence of sign of drunkenness. Blood alcohol concentration measured as gms alcohol/100mls blood.

^ Calculation of P value uses chi-square for trend (df =1)
